# *Lyonia ovalifolia* (Angeri) poisoning: A case report

**DOI:** 10.1002/ccr3.6128

**Published:** 2022-07-25

**Authors:** Niraj Kumar Sharma, Madhur Bhattarai, Sangam Shah, Pawan Gyawali, Kushal Baral, Hemanta Banstola

**Affiliations:** ^1^ Institute of Medicine Tribhuvan University Maharajgunj Nepal

**Keywords:** angeri, *Lyonia ovalifolia*, poisoning

## Abstract

*Lyonia ovalifolia* (angeri) is a deciduous tree whose shoot and leaves are toxic. Its chemical constituents include grayanane diterpenoids, lyoniol A, and other toxic compounds. Young children might consume it intentionally or unintentionally, with subsequent adverse health outcomes and even mortality depending on the amount ingested. We present a case of an adolescent girl who developed poisoning on ingestion of angeri leaves.

## Abstract 2

Adequate information about the toxic plants and their potential toxic effects and outcomes must be given to parents, adolescents, and children. Recognition of symptoms and their timely management is crucial in preventing potential health hazard.

## 1 INTRODUCTION

Acute poisoning accounts for significant morbidity and mortality among children and adolescents in developing countries like Nepal.^1^ As per WHO, acute poisoning is attributed to an estimated 45,000 deaths and an incidence of 1.8 in 100 thousand children and adolescents under the age of 20 years worldwide.^2^ Nepal, being a country of rich biodiversity, there are abundant medicinal herbs and plants. However, some of them are potentially toxic. Inadequate knowledge and ignorance about these toxic medicinal plants could lead to severe accidental poisoning.^3^

**FIGURE 1:**
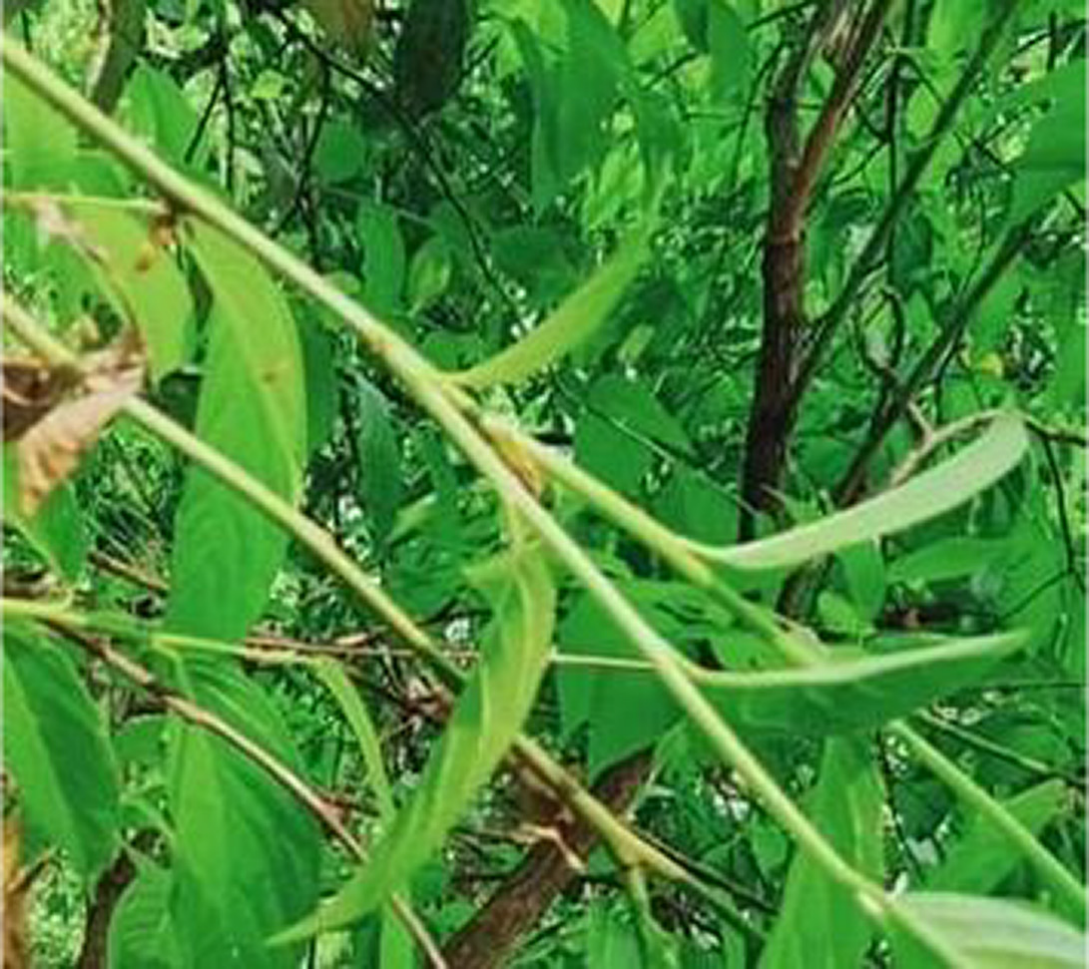
Leaves of Angeri plant

*Lyonia ovalifolia* is a deciduous tree or shrub distributed in hilly and valley areas of Nepal China, Taiwan, Northern India, and Thailand. It is called Angeri in the local language. (Figure 1) The young leaf and shoot of *L. ovalifolia* are toxic whose consumption by cattle or humans can cause vomiting, dizziness, and generalized weakness and may induce the paralysis of motor nerve terminals. The incidence of angeri poisoning in cattle is frequently observed especially in goats. Sometimes children unintentionally consume toxic parts causing poisoning.

## 2 CASE PRESENTATION

A 16‐year‐old girl with no significant medical history presented to our emergency department with complaints of dizziness, generalized weakness, and multiple episodes of vomiting for 3 h. On further enquiry, patient party mentioned that she had accidentally ingested 3–4 leaves of angeri plant on her way to school around 20 h ago. No other substance use, drug history, and known allergy to drug or food were confirmed. She had no significant complaints other than an unpleasant taste sensation immediately after ingestion and complained only of mild disturbance in sleep in the first 20 h.

**FIGURE 2:**
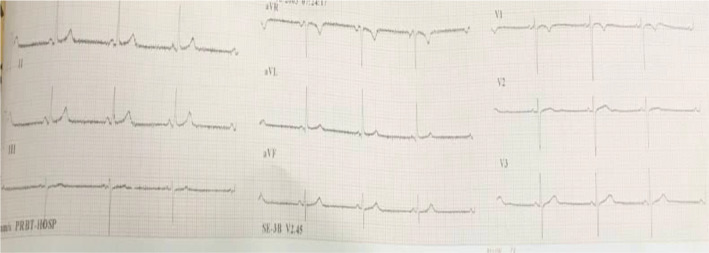
Electrocardiogram of the patient showing sinus bradycardia

On examination, the patient was ill‐looking but was well oriented to time, place, and person. Her blood pressure was 60/40 mm of Hg, pulse rate of 44 beats/min, regular and normal volume, temperature 98.8 F, and respiratory rate of 18 breaths/min. Oxygen saturation was 97% at room air. The electrocardiogram finding showed sinus btadycardia. (Figure 2) She was immediately resuscitated with one pint of intravenous normal saline stat, intravenous (IV) ondansetron 4 mg stat, and IV atropine 0.6 mg stat. After resuscitation, her blood pressure reached 80/50 mm of Hg with pulse rate of 65 beats/min. She was kept under close monitoring with IV fluids; and IV atropine was administered for bradycardia (heart rate <50 beats/min).

Laboratory investigations revealed total count of 6700/mm^3^, platelets 212,000/mm^3^, hemoglobin 11.3 gm%, random blood sugar 81 mg/dl, urea 25 mg%, creatinine 1.1 mg%, and normal liver function tests.

Her blood pressure and pulse rate after 12 h of admission were gradually maintained at 90/60 mm of Hg and 85 beats/ min, respectively. No other dosage of atropine was administered. After 24 h of constant monitoring and observation, she was discharged with stable vitals and she was advised to follow up in 2 weeks and earlier in case of emergence of any symptoms. On follow‐up after 2 weeks, she had no fresh issues and was improving.

## 3 DISCUSSION

*Lyonia ovalifolia*, belonging to the family Ericaceae, is a deciduous shrub that can grow up to 4 meters tall. It is native to the Himalayas, Nepal, China, India, Japan, Cambodia, Myanmar, Thailand, Vietnam, and Malaysia. It is known as Angeri in Nepal, Anyaar in India, and Nejiki in Japan.^4^, ^5^ In the rural parts of Nepal, Angeri is used as fodder for livestock. Mature leaves do not cause poisoning allowing farmers to use them as fodder for domestic animals in rural parts of Nepal. However, the young leaves and buds are toxic. So, when cattle are accidentally fed young shoots and leaves as fodder, it could lead to poisoning. Cattle may also consume young shoot themselves while taken to the jungle for grazing and suffer generalized weakness, vomiting, fever, and even death.^3^ Children might also accidentally ingest toxic parts of the plant, leading to morbidity and even death. It is also used to cure wounds, burns, and scabies by traditional medicine practitioners in Nepal, which also defines another potential source of poisoning.

The patient had no known allergy to drug or food. Also, she had no symptoms for 20 h after ingestion of leaves and presented to our center after 4 h of development of symptoms. While presenting to us, she had no shortness of breath, wheezing, and angioedema. All these sorts of presentation are less likely in cases of anaphylaxis. The major class of compounds reported in this plant are alkaloids. Various compounds such as lyoniol‐A, secorhodomollolides A and D, grayanane, lyoniol D, hexacosane, hexacosanol, sitosterol, taxaxerol, lyonin A‐C, lyoniside, lyonireinol, ovfolinins A‐E, lyonitoxin, quercetin 3‐ galactoside, apigenin, luteolin, quercetin, and epicatechin have been isolated from this plant.^6^ Grayanane diterpenoids are exclusively found in plants of Ericaceae family and are, therefore, considered a chemotaxonomic marker of the family. They have a specialized carbon skeleton with highly oxygenated functionalities. These are extremely toxic to mammals, especially to the heart, and nervous system. The plant exhibits diverse biological properties as analgesic, anti‐inflammatory, antimicrobial, antioxidant, anti‐cancer, and antiviral.^6^, ^7^ This could be partly attributed to its CAMP regulatory and protein tyrosine phosphatase 1B (PTB1B) activity. Further extensive research is warranted to explore its potential benefits. On the contrary, it has an insecticidal action due to its potential toxic effects. Lyoniol A is also a toxic component that causes marked excitation of vagal afferent nerve causing respiratory depression, hypotension, muscle tremor, and relief of the decerebrate rigidity.^8^ It may induce the paralysis of nerve centers and motor nerve terminals.^6^ In our report also, the patient is presenting with dizziness, generalized weakness, vomiting, hypotension, and bradycardia, which are explained by the action of these toxic compounds.

Management of plant poisoning is usually supportive care and symptomatic treatment, as done in this case.^9^ The specific antidote of angeri poison is not available in the literature. Thus, prevention is of key importance. Children, adolescents, and parents must be given ample information about the toxic plants and counseled about the potential risk of poisoning from such toxic plants. Early identification of poisoning based on history or witness of ingestion and recognition of symptoms could help in timely seeking of medical care, thereby reducing its deleterious effects.

## 4 CONCLUSION

The young shoot and leaves of *L. ovalifolia* (angeri) are poisonous. It is possible that children and young adolescents can consume it unintentionally and may present with variable symptoms. Timely symptomatic management and careful observation is warranted. Children, adolescents, and parents of the targeted area should be given adequate information and counseled about the potential poisonous plant and their toxic effects.

## AUTHOR CONTRIBUTIONS

SS and MB wrote the original manuscript and reviewed and edited the original manuscript. NKS, KB, PG, and HB reviewed and edited the original manuscript.

## CONFLICT OF INTEREST

The authors have no conflict of interest to declare.

### ETHICAL APPROVAL

None.

### CONSENT

Published with the written informed consent of the patient.

## Data Availability

All the required information is in the manuscript itself.

## References

[1] Acharya K , Kandel IS , Gupta S , Poudel SD . A study on incidence and patterns of acute poisoning cases in an emergency Department of Western Region of Nepal. J Gandaki Med Coll. 2019;12(2):59‐62.

[2] Sminkey L . World report on child injury prevention. Inj Prev. 2008;14:69. 18245322 10.1136/ip.2007.018143

[3] Ghimire SK , Jha PK , Karmacharya SB , Chettri MK , Thapa CB , Shrestha BB , et al. Medicinal Plants in Nepal: an Anthology of Contemporary Research. ResearchgateNet; 2008. https://lib.icimod.org/record/6883. Accessed 2022 Jun 25.

[4] *Lyonia ovalifolia* ‐ Trees and shrubs online. https://treesandshrubsonline.org/articles/lyonia/lyonia‐ovalifolia/. Accessed 2022 Jun 25.

[5] *Lyonia ovalifolia* ‐ Useful temperate plants. http://temperate.theferns.info/plant/Lyonia+ovalifolia. Accessed 2022 Jun 25.

[6] Sahu N , Arya KR . Ethnobotanical and ethnopharmacological activities of *Artemisia nilagirica*, *Lyonia ovalifolia*, *Sarcococca saligna* and *Taraxacum officinale* . 2017;8(11):4818‐4825.

[7] Li CH , Zhang JY , Zhang XY , Li SH , Gao JM . An overview of grayanane diterpenoids and their biological activities from the Ericaceae family in the last seven years. Eur J Med Chem. 2019;166:400‐416. 30739823 10.1016/j.ejmech.2019.01.079

[8] Ono H , Fukuda H , Kudo Y . Excitation by lyoniol‐a of vagal afferent nerves and the reflex autonomic and somatic actions in rats. J Pharmacobiodyn. 1981;4(12):940‐946. 7341750 10.1248/bpb1978.4.940

[9] Eddleston M , Persson H . Acute plant poisoning and antitoxin antibodies. J Toxicol Clin Toxicol. 2003;41(3):309‐315. 12807314 10.1081/clt-120021116 PMC1950598

